# Study protocol to evaluate an integrated intervention using a pill ingestible sensor system to trigger actions on multifaceted social and behavioral determinants of health among PLWH: an open-label, usual care-controlled, randomized trial

**DOI:** 10.1186/s12889-025-24909-0

**Published:** 2025-10-31

**Authors:** Honghu Liu, Jie Shen, Yan Wang, Tony C. Carnes, Alison B. Hamilton, Mallory D. Witt, Eric S. Daar

**Affiliations:** 1 Section of Public and Population Health, School of Dentistry, University of California, Los Angeles, CA USA; 2 etectRx Inc, Gainesville, FL USA; 3 David Geffen School of Medicine, University of California, Los Angeles, CA USA; 4 VA Greater Los Angeles Healthcare System, Los Angeles, CA USA; 5 Department of Medicine, Division of HIV Medicine, Lundquist Institute at Harbor-UCLA Medical Center, Torrance, CA USA

**Keywords:** HIV, Pill ingestible sensor, Social and behavioral determinants of health, Adherence to antiretroviral therapy, Intervention

## Abstract

**Background:**

A pillar in the effort to end the HIV epidemic, the “Undetectable equals Untransmittable” (U = U) campaign has led to reduced HIV stigma and empowered people living with HIV (PLWH). Viral suppression, which eliminates risk of sexual HIV transmission, highlights the crucial role of adherence to antiretroviral therapy (ART). Strategies to enhance adherence have primarily focused on pill-taking reminders, often without real-time monitoring or interventions to address social and behavioral determinants of health (SBDOH). Suboptimal adherence to antiretroviral therapy often requires interventions from multidisciplinary teams, including nurses, social workers, and case managers to address SBDOH. These interventions often only occur weeks to months after adherence problems are identified, leading to delays in addressing patient needs and adherence to ART.

**Methods:**

In this protocol paper, we describe the design and procedures for a National Institutes of Health-funded randomized controlled trial that tests an innovative, integrated intervention combining the latest pill ingestible sensor system (the ID-Cap from etectRx) and strategies to address SBDOH. The intervention will trigger real-time actions to rapidly address SBDOH-related barriers when pre-specified patterns of poor adherence are detected (antiretroviral doses missed on three days within a five-day period). A cohort of 110 PLWH who have, or are at high risk for, sub-optimal adherence will be recruited from an HIV clinic in Los Angeles County. Participants will be randomized into the ingestible sensor intervention arm or the usual care arm. The intervention will run for 16 weeks, followed by a 12-week post-intervention period to evaluate sustainability. The primary outcomes are acceptability of the integrated intervention, frequency and timeliness of SBDOH intervention, level of challenges of SBDOH in HIV treatment, adherence to ART measured by ID-Cap system, and self-reported medication adherence. Secondary outcomes are plasma HIV viral load and high risk sexual activity,

**Discussion:**

Real-time monitoring of adherence will enable multidisciplinary teams to promptly address difficulties of SBDOH with tailored content to fit each individual’s unique challenges and needs. When completed, this trial will define the potential role of ingestible sensor system in a comprehensive program to address barriers to adherence and improve control of the HIV epidemic.

**Trial registration number:**

NCT06480578. Date registration: June 28, 2024.

**Supplementary Information:**

The online version contains supplementary material available at 10.1186/s12889-025-24909-0.

## Background

People living with HIV (PLWH) can have long and healthy lives by taking antiretroviral medications as prescribed and maintaining undetectable viral load [1–3]. Undetectable equals Untransmittable (U = U) is firmly established in science, meaning that those adhering to antiretroviral therapy (ART) and achieving and maintaining an undetectable viral load cannot transmit HIV through sexual behaviors to others [4]. Thus, treatment as prevention is a key pillar of the Ending the HIV epidemic in the US by 2030. U = U also reduces HIV stigma, which has contributed to health inequalities and remains a barrier to effective HIV care [3].

In Healthy People 2030, the Centers for Disease Control and Prevention (CDC) recognize five key areas of social determinants of health (SDOH): economic stability, education access and quality, health care access and quality, neighborhood and environment building, and social and community context [5]. The relationship between SDOH and HIV care outcomes of adherence and viral suppression are also well recognized [6–10]. Behavior determinants, such as substance use and sexual risk behaviors, are also important components of the HIV context, and social and behavioral determinants of health (SBDOH) intersect [11] which influence the level of adherence to ART. In a cross-sectional survey conducted in 2015–2019 [6], an estimated 83% of PLWH reported issues with at least one SBDOH indicator. Adverse SBDOH, especially food insecurity, housing instability, poverty, lack of social support, and risky behaviors are significantly associated with low medication adherence [12]. People who had problems with more than four SBDOH indicators were 31% less likely to have optimal adherence to ART in the past month and 20% less likely to have durable viral suppression in the past year [6]. It is therefore essential to develop effective and timely interventions that address SBDOH in order to end HIV epidemic in the community where people “grow, live, work and age” [8].

Extensive research has been done on the measurement of adherence to ART in the past two decades [13–15], such as directly observed therapy (DOT) [16], self-report [17], pharmacy refill [18], pill-box [19], medication event monitoring system (MEMS) [13, 20], Wisepill [14], as well as dry blood spot [21], hair [22] and plasma [23] concentration. Among the different adherence measures, ingestible sensor technology is the most recent method that can provide a real time, non-inferred measure of pill ingestion [24]. Real-time monitoring of adherence, accompanied with text message reminders, can facilitate and support the development of an effective intervention program [25, 26].

Most adherence interventions lack the incorporation or consideration of SBDOH. Instead, the majority of these interventions focus on improving patient education, regimen management, and frequent monitoring through reminder messages [12]. Improving adherence to ART still remains a global challenge due to no gold-standard methods for adherence measurements or the absence of well-established interventions to enhance medication-taking behavior [26]. In addition, it is critical to understand the broader context of poor adherence in PLWH and design interventions that go beyond monitoring and reminding to address the underlying reasons for nonadherence, such as an adverse SBDOH. Thus, in this study, we will integrate strategies to address SBDOH with an ingestible sensor system (ISS) that can objectively measure, monitor, and intervene, in real-time, to address suboptimal antiretroviral (ARV) adherence [24].

We have demonstrated feasibility, acceptability and utility of an ingestion sensor system that monitored and automatically provided text message reminders [24]. In this study,. The current paper describes the protocol for a randomized controlled trial to implement this ingestible sensor triggered intervention.

## Methods

### Aims and hypotheses

The overarching goals of this study are to develop and test a targeted program that integrates the cutting-edge ingestible sensor system that produces non-inferred measures of adherence, with an interest in addressing SBDOH to improve adherence and viral suppression. This trial has the following aims: (1) Evaluate the acceptability, frequency and timeliness of SBDOH intervention, and level of challenges of SBDOH in HIV treatment among PLWH; (2) evaluate the efficacy of the integrated intervention of ISS-SBDOH for monitoring, facilitating and improving adherence to ART; and (3) evaluate the efficacy of the ISS-SBDOH intervention for improving virologic outcome and reducing high risk sexual activity and STIs. We hypothesize that an integrated intervention program that combines the usage of accurate ingestible sensor system with real-time reminders and that triggers interventions to address issues of SBDOH by nurses, case managers, and social workers from the same clinic where the participants receive care will have a synergistic effect on enhancing adherence to ART and reducing HIV risk-taking behavior.

### Study design and setting

**Fig. 1:**
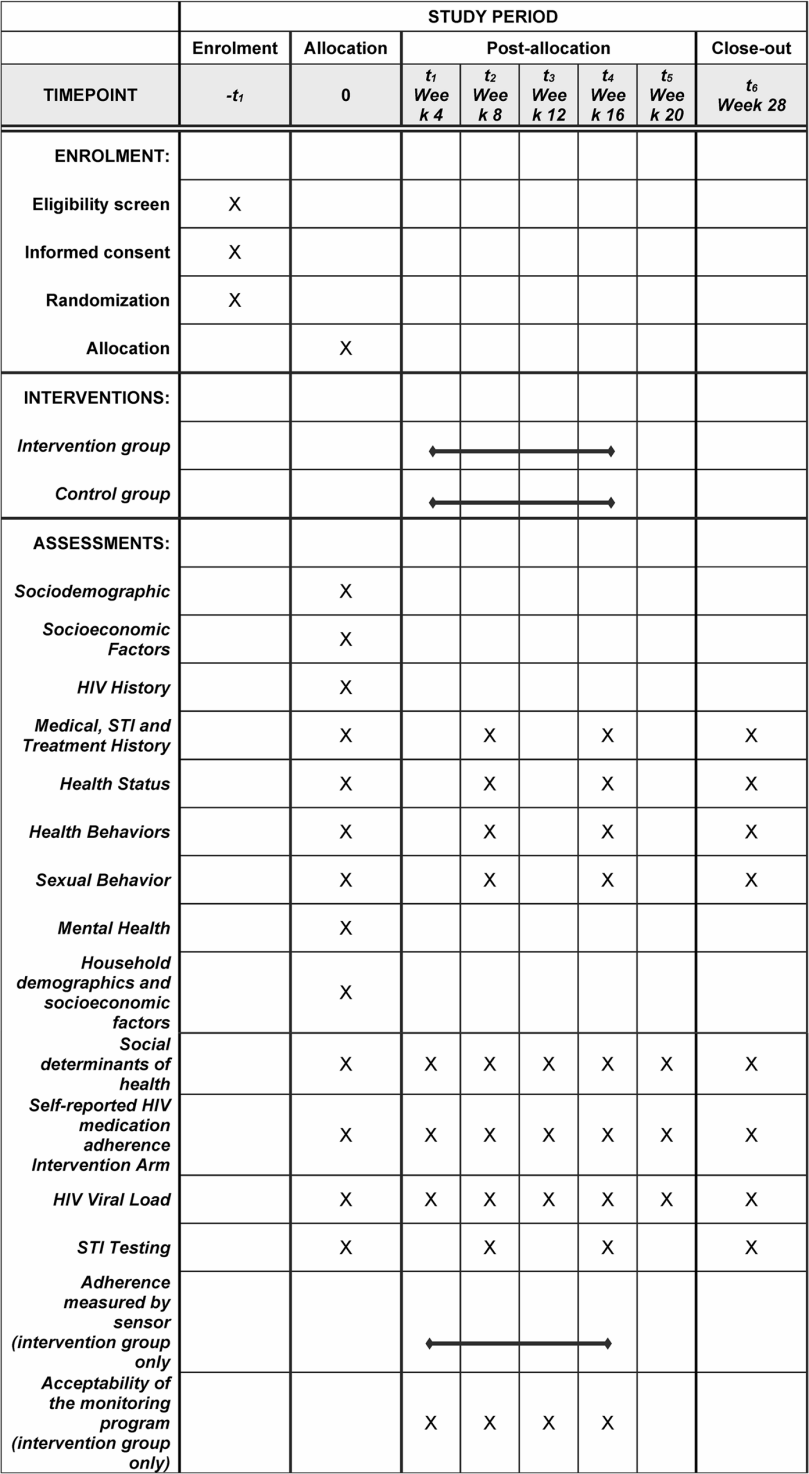
SPIRIT figure for the trial Medical. STI and Treatment History

Using real-time monitoring, the integrated intervention—ingestible sensor system and SBDOH (ISS-SBDOH)—will be able to immediately trigger the existing clinic multidisciplinary team to address SBDOH issues when predefined patterns of suboptimal adherence are detected (antiretroviral doses missed on three days within a five-day period). The SPIRIT figure of this trial, including the schedule of screening, enrollment and follow up visits, is illustrated in Fig. 1.

**Fig. 2:**
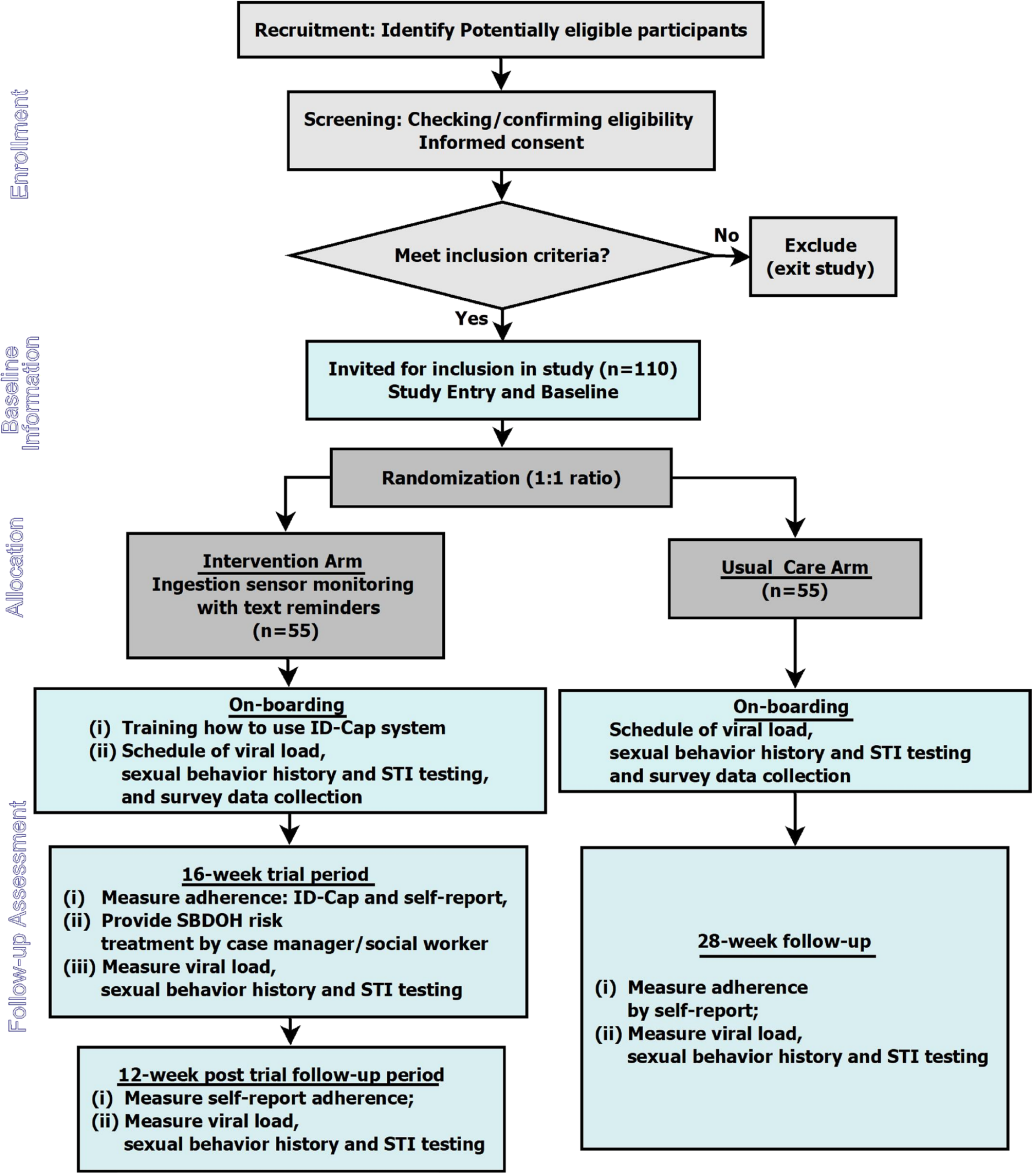
Overview of Trial Flow

The overall flow of the study is shown in Fig. 2. This study is an open-label unblinded randomised control trial. Participants will be randomly assigned by 1:1 allocation to either: (1) the ISS-SBDOH arm, which will include training on how to use the ingestible sensor system (the ID-Cap from etectRx), data collection procedures, and schedules; or (2) the Usual Care (UC) arm, which will not use of ID-Cap system. On-boarding procedures will be done for both study arms. In the first 20 weeks, we will collect data on primary end points of acceptability of the ISS-SBDOH intervention, frequency and timeliness of SBDOH intervention, level of challenges of SBDOH in HIV treatment, and adherence to ART; the secondary end points will include viral load, high risk sexual activity and frequency of detectable sexually transmitted infections (STIs); and other measures. Durability of any observed effects from the ISS-SBDOH intervention will be evaluated during a 12-week post-ID-Cap system follow-up period. The trial will be conducted in accordance with the guidelines for Good Clinical Practice (GCP) and will satisfy Consolidated Standards of Reporting Trials (CONSORT) reporting conventions [27].

### Study duration

The 16-week intervention phase is long enough to evaluate the acceptability and efficacy of the integrated ISS-SBDOH intervention and fatigue of using ISS [28, 29]. SBDOH treatment sessions may not be able to completely resolve all the adverse SBDOH issues a patient has, but will rapidly start to work out issues With 16-weeks of ID-Cap, the trial will be able to potentially trigger multiple SBDOH intervention sessions for a given participant, and provide rich data to evaluate the effect of the integrated intervention. The 12-week post ID-Cap intervention period is also long enough to detect rapid dissipation of any effects of using the ID-Cap system and the timely intervention on SBDOH triggered by the ID-Cap system. Although there will be no SBDOH intervention triggered by dose missing patterns during this 12-week period, study participants can still get SBDOH interventions through regular clinic visits, provider contacts and/or participant-initiated phone calls.

### Eligibility criteria for participants

Table 1 presented the inclusion and exclusion criteria for this study. The enrollment criteria were chosen to enroll participants who would be referred to such a system because of suspected adherence difficulties.

**Table 1:** Eligibility criteria

Inclusion Criteria	Exclusion Criteria
• HIV-infected individuals in HIV care • Greater than 17 years of age • Demonstrated ability to take co-encapsulated ARVs at time of screening; able to provide informed consent. • On ART with sub-optimal adherence estimated by either patient (self-reports < 90% adherence over last 28 days) or treating clinician (e.g., based on gaps in treatment (e.g., missed appointments) or viral load elevations within last 6 months), or at high risk for sub-optimal adherence, or with known challenges with SBDOH (e.g., unstable housing, substance use disorder, unstable housing, and poverty).	• Unable to provide informed consent due to severe mental or physical illness, cognitive impairment, or substance intoxication. • Pregnancy or inability to follow the study procedures manifested during the intake, as evidenced by mental confusion, disorganization, intoxication, withdrawal, risky or threatening behavior.

### Participant recruitment

Target populations will be those PLWH who have or are at high risk for sub-optimal adherence to ART as defined in the inclusion/exclusion criteria (Table 1). Study subjects will be recruited through the LA County safety net HIV clinic at Harbor-UCLA Medical Center through its Lundquist Institute. Both active and passive recruitment approaches will be used: (i) Clinic-wide information sessions will be conducted by the research team on a regular basis; (ii) Posters, flyers, and brochures will be posted and readily available in each clinic; (iii) Providers will be asked to review charts and appointment schedules in order to identify potential participants and ask about their interest in the study. Clinic physicians and staff will discuss the study with eligible patients and refer them to the study staff for enrollment. Research staff will be available for initial screening and will adapt schedules to enroll patients after clinic visits.

### Informed consent and enrollment

Once eligibility is confirmed, trained study staff will conduct the informed consent process in a private setting. This will involve presenting a consent document that outlines the study’s rationale, procedures, potential risks and benefits, confidentiality measures, and the participants’ rights and responsibilities. Individuals who agree to participate will provide their consent by electronically signing the informed consent form.

### Randomization

Randomization will take place after the baseline assessment and before the intervention begins. The UC arm is chosen as the control condition because it meets ethical and moral requirements to attempt treatment. Eligible patients will be randomized to one of the two conditions using a stratified urn randomization procedure to increase the likelihood of balanced allocation of prognostic variables at baseline. We will stratify by two factors: (i) single/multiple tablet regimen, and (ii) detectable/undetectable viral load at baseline through a 2 × 2 factorial stratification, to assign them into intervention arm or UC arm within each of the four cells. Randomization is implemented electronically through REDCap.

### On-boarding for the study

All study participants, including both ISS-SBDOH and UC arms, will go through an on-boarding process to be trained and/or taught about study procedures and data collection schedules (e.g., the date/time for measurements on plasma HIV viral load, STI testing (frequency of detectable STIs), and survey data collection). Study participants will be informed of the schedule of events and assessment administration. For the ISS-SBDOH arm, participants will be trained on how to use the ID-Cap ingestible sensor system, including use of wristband (or necklace, if a patient prefers); how to download and install app in iOS and/or Android platforms; the basic features, functions, and nomenclature of the ingestible sensor and patient app. An ID-Cap system is provided, along with supplemental test equipment (e.g., laptops for training videos) for the training. Patient training materials and information provided include training videos and presentations, quick start guides, and user manuals. Training videos cover the following topics: overview of the ID-Cap system, ID-Cap app setup and use, setup of charger and reader with app and system use, routine use of the system with use of the patient app, and app navigation and functionality, system messages and meaning, and where to get help. At the end of a training session, the participants will be asked to demonstrate the steps for completing a successful ingestion using the reader and patient app. The training materials and device instruction for use are designed to support a self-guided supplemental training program. All training and on-boarding processes will take place at office facilities of Lundquist Institute at Harbor-UCLA Medical Center.

### Customized pre- and post-dose reminder

We will use a secure and confidential web-based interface to send a short message service (SMS) text when a dose has not been ingested within a pre-determined time, informing the participant that a dose was missed. We will customize with patients the schedule of messages (i.e., what window qualifies as a missed dose, and how soon after a dose is missed a text message would be sent), and customize the content of the text messages to fit each individual’s needs and preferences. In addition to missed dose reminder (post-dose reminder), we will also add a pre-dose reminder that will be sent to each participant who missed a dose the previous day.

### SBDOH Intervention

**Fig. 3:**
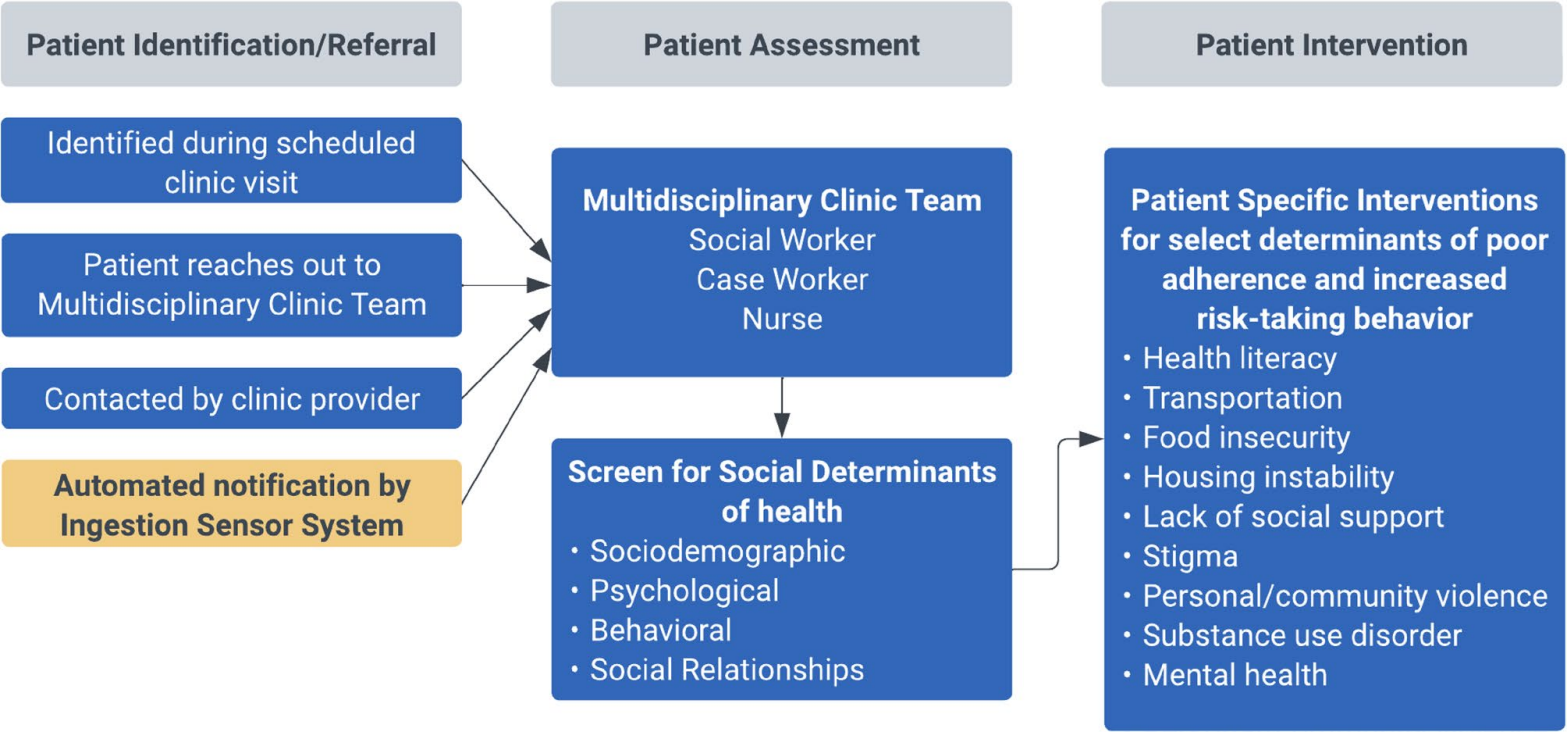
Program for addressing social and behavioral determinants of health in high risk patients

Like many urban safety net HIV clinics, enrollment clinical site personnel (nurses, case workers, and social workers) can provide services for SBDOH needs by a multidisciplinary team. Figure 3 summarizes how this service can be activated in the intervention arm. In UC arm, patient identification/referral is initiated during a clinic visit, or if a patient reaches out between visits to their primary provider or a member of the multidisciplinary team. Once triggered, the team will meet with the participant to assess SBDOH and use resources and expertise available to address key issues. The team members are experienced and when appropriate can provide referrals to dedicated HIV mental health specialists. The social workers and case managers are experienced at addressing social/behavioral determinants, including but not limited to in- or out-patient substance use disorder, housing, and food insecurity. The mental health specialists are also focused on and experienced in addressing the challenges PLWH encounter, including anxiety, primary psychiatric conditions, as well as stigma and discrimination. However, this signal to address SBDOH needs often occurs weeks to months after issues have emerged. In this study, the SBDOH intervention will be triggered if a participant has missed his/her prescribed ARVs for a total of 3 days within a five-day period monitored by real-time sensor. In addition, during the first 16 weeks, the participants in the intervention group will use ID-Cap to monitor their daily HIV adherence in real-time and receive their pre- and post- dosage reminders. With 4-week intervals, we will ask participants for their feedback on ID-Cap and/or the SBDOH session, when applicable.

### Self-Reported SBDOH needs

In 2013, the Institute of Medicine (IOM) established a committee to develop an initial 25-item questionnaire to measure SBDOH in 2017 [30]. Based IOM’s scale, Menza et al. in 2021 created a 10-item index of SDOH in the context of HIV care outcomes, including missed appointments, adherence, and viral suppression [6]. We will extend this questionnaire by both counts of social determinants and the degree to which the determinants may impact health outcomes (e.g., low/medium/high).

### Schedule of events and assessments

A research assistant will complete the baseline assessments within 30 days of screening using computer-based and/or paper-based assessments. Responses will be directly entered into a computer database by the research assistant. Figure 1 summarizes the event and assessment administration schedule.

Study participants will be asked to complete the eligibility form, informed consent, and consent to contact with providers to determine current medication regimen and determine if the patient meets suboptimal adherence criteria. They will also be given an over-encapsulated pill to assure it is tolerable. The following multilevel individual and social characteristics will be collected. Individual Characteristics include sociodemographic (age, gender identity, race/ethnicity), socioeconomic factors (income, education, occupation, marital status, children); HIV history (years since HIV diagnosis, years on HIV treatment, current HIV regimen, overall pill burden, and disease comorbidity); medical, STI and treatment history; health status (SF-12 Short Form questionnaire no [31]); health behaviors – alcohol (number of alcoholic beverages consumed per day and week using NIDA cutoffs by gender [32]) and substance use; sexual orientation (classified as homosexual, bisexual, heterosexual or uncertain); sexual behavior (number of male and female partners, condom use, and unprotected anal/oral sex). Social Characteristics include household demographics and socioeconomic factors (family size, dependents, family income, housing stability), Household Food Insecurity (Short Form Household Food Security Scale [33]), and 10-item index of SDOH [6].

During the first 16-weeks, ART adherence of the ISS-SBDOH arm will be measured by ID-Cap and self-report, and adherence of the UC arm will be measured only by self-report. After 20 weeks there will be a post ID-Cap follow-up period where all subjects will be followed for an additional 10 weeks during which adherence of both arms will be measured by self-report as outlined in Fig. 1.

## Outcomes

### Primary outcome

(i) Acceptability of the integrated intervention will be measured at baseline, and around week 4, 8, 12, and 16, evaluated by quantitative measures interviews. The items in the quantitative measure will be rated on a five-point Likert scale ranging from very strongly disagree to very strongly agree. Acceptability will be measures in terms of: overall satisfaction (would recommend to friend, would use outside of study setting, satisfied with system), utility, and specific items such as helpfulness and convenience. In addition, we will ask participants to rate items specific to the ID-Cap system (comfort of wristband, comfort receiving text messages).
(ii) Frequency and timeliness of SBDOH intervention, level of challenges of SBDOH in HIV treatment will be measured at baseline, and around week 4, 8, 12, 16, 20, and 28. We will collect SBDOH measures, such as economic stability (e.g., food security), health (e.g., gap in health coverage), neighborhood and built environment (e.g. transportation needs), social and community context (e.g., violence, and criminal justice involvement), and substance and alcohol use, sexual behavior, and other health related behaviors. Challenges of SBDOH will be measured as the count of SBDOH issues one is facing, and the degree of each issue (e.g., low/medium/high with housing challenge determined by nurse or care coordinator) with summary scores. Frequency and timeliness of SBDOH intervention are measured as number of sessions, and time to first, and subsequent sessions, if applicable, from baseline, and others. This information will also be collected when intervention is triggered by the monitoring system.
(iii) Adherence to ART measured by ID-Cap system for 16 weeks.
(iv) Self-Reported Medication Adherence [34]. We will use a widely used measure of percent of prescribed dose taken during the preceding seven days. We will also measure patterns of dosing (e.g., patterns of consecutive missed doses in the past two weeks).

### Secondary outcomes

(xxii) Plasma HIV viral load will be measured at baseline, and at weeks 4, 8. 12, 16, 20, 28 thereafter.
(xxiii) High risk sexual activity will be assessed by survey of number of sexual partners, condomless sex, and screening of all orifices for chlamydia, gonorrhea and blood for syphilis.

### Process measures

(vii) Participants’ feedback will be measured on ID-Cap at week 4, 8, 12, 16 and 20, and SBDOH intervention at weeks 4, 8, 12, 16, 20 and 28.

### Statistical analyses

We will use α < 0.05 as the significance level for statistical testing, and will use appropriate corrections (e.g., Bonferroni correction) for multiple comparisons to ensure that the effective type I error remains at 0.05 level. Statistical analyses will be conducted on an intention-to-treat sample using the SAS and R statistical software.

### AIM 1. Evaluate the acceptability, frequency and timeliness of SBDOH intervention, and level of challenges of SBDOH in HIV treatment among PLWH

We hypothesize that greater than 75% of the participants in the ISS-SBDOH arm will rate the intervention as fitting their routine activities of daily life. To test this hypothesis, we will perform analyses in two levels: (1) Obtain point estimates of ratings about the intervention and conduct one group binomial test of the proportion with answers that indicates a fit (or better fit). This will be done at each of the time point of baseline, and weeks 4, 8, 12, and 16; (2) Generalized linear mixed models (GLMM) will be used to model the changes of ratings over time controlling characteristics (e.g., demographics, SBDOH, regimen type (single/multiple pill), and viral load). GLMM can model not only global fixed effects (e.g., the regimen type), but also random effects (e.g., change over time within a person). We also hypothesize that the ISS-SBDOH arm will have significantly higher frequency and better timeliness of the SBDOH intervention sessions than the UC arm; and the ISS-SBDOH arm will have lower level of challenges of SBDOH over time than the UC arm. To test this, we will use GLMM to model the number of challenges of SBDOH over time through parameter estimate of time (fix effect) in the model.

### AIM 2. Evaluate the efficacy of the integrated intervention of ISS-SBDOH for monitoring, facilitating and improving adherence to ART

We hypothesize that the ISS-SBDOH arm will have significantly higher mean adherence to ART than the UC arm. GLMM will be used modeling adherence to ART across the contact periods. An arm (ISS-SBDOH or UC) dummy variable will be included in the model to test if the level of adherence is different between the two arms. If the distributions are substantially skewed, we will evaluate categorized clinical cut-off values using generalized estimating equation (GEE) analysis.^95^ For possible non-linear relationships between outcomes and time, we will use generalized additive mixed model (GAMM). The trend of adherence changes before and after 16 weeks will be compared. In addition, we will evaluate group differences in demographics (e.g., gender) and control for these variables in modeling. We also hypothesize that the use of ISS-SBDOH will be associated with lower levels of non-adherence (e.g., fewer single missed doses, a shorter maximum duration of taking no doses) than the UC arm over time. We will make full use of machine learning and data mining methods, including classification and regression trees (CART), random forests, XGBoost, Generalized Additive Models, to identify non-adherence patterns in the data. We will use GLMM/GAMM to model adherence between ISS-SBDOH and UC arm over time to determine if it differs between arms. The GAMM modeling will graphically show the change trend over time. We will also evaluate the distribution of DTEs and their changes over time in the ISS-SBDOH arm. We hypothesize that the differences in adherence between the ISS-SBDOH and the UC arms will be sustained during the 10-week post-intervention period. GLMM/GEE and GAMM will be used to model adherence between the two arms, including parameterization that can distinguish the 16-week trial and 12-week follow-up periods.

### AIM 3. Evaluate the efficacy of the ISS-SBDOH intervention for improving virologic outcome and reducing high risk sexual activity and STIs

We hypothesize that the participants in the ISS-SBDOH arm will have a low level of viral load and reduced high-risk self-reported sexual activity and frequency of detectable STIs compared to the UC arm. GLMM and GAMM will be used to model viral load of patients and frequency of detectable STIs in the ISS-SBDOH arm and the UC arm across the contact periods (baseline and follow-ups) to evaluate an $\:arm\times\:time$ interaction. We also hypothesize that the differences in viral load, and level of high-risk sexual activity and frequency of detectable STIs between the ISS-SBDOH and the UC arms will be sustained during the 12-week post-integrated intervention follow-up period. GLMM/GEE and GAMM will be used to model the viral load, level of high-risk sexual activity, and frequency of detectable STIs between the two arms including parameterization that can distinguish the 16-week intervention phase and 12-week sustainability phase.

### Sample size calculation

Sample size calculation and power analysis were conducted based on the primary and secondary end points. For Aim 1, 55 subjects to be recruited in the ISS-SBDOH arm and 55 in the UC arm enable us to obtain stable point estimates and test the hypotheses for acceptability of the intervention program using one sample binomial approach. For the frequency and timeliness of SBDOH intervention and level of challenges, group sample sizes of 55 in the ISS-SBDOH arm and 55 in the UC arm will enable us to detect a difference as small as 0.5 in a continuous outcome (a medium Cohen’s effect size) with 80% power, and a significance level of 0.05 using a two-sided two-sample t-test. For Aim 2, using repeated measures analysis with intra-subject correlation of 0.1, a type I error of 0.05, a type II error of 0.2 (or 80% power), and average number of available data points 3.5, out of 5 data points (baseline, weeks 4, 8, 12, 16), 55 subjects in each of the two arms will enable us to detect a difference as small as 0.29 in standardized unit of the outcomes (e.g., adherence to ART) between the two arms, which is statistically and clinically meaningful. For Aim 3, for the binary outcome variable of viral suppression, using repeated measures analysis with similar set-up as Aim 2, the sample size of 55 participants in each of the 2 arms will be able to detect a 9% difference (or an odds ratio of 2.7) between the 2 arms (assuming 85% viral suppression at week 16 for control arm) and this effect size is considered clinically meaningful. Thus, the planned sample size should be sufficient for the proposed analyses.

### Data collection and management

All survey data are entered directly into REDCap, a HIPAA-compliant data management system, and securely stored on the University of California, Los Angeles (UCLA) Health Box, a password-protected network. Each participant is assigned a unique study number upon consent, with all data de-identified for monitoring and audits to ensure confidentiality, and for presentation or publication of results. Study team members receive training in confidentiality and ethical research practices and sign confidentiality agreements. Investigators report unforeseen issues, safety concerns, or adverse events to the UCLA institutional review board (IRB) promptly, with protocol modifications reviewed and approved as needed.

As a low-risk behavioral intervention, the study does not include stopping rules or interim analyses. An independent Data Safety Monitoring Board reviews progress, safety, and adverse events biannually. Safety and progress reports are submitted to the IRB and the funder at least annually.

## Discussion

Utilizing real-time monitoring facilitated by the advanced ID-Cap ISS, the integrated intervention—comprising the ingestible sensor system and SBDOH—can promptly activate the clinic’s existing multidisciplinary team upon detection of predefined patterns indicative of suboptimal adherence. This approach effectively addresses challenges related to SBDOH associated with poor adherence in a timely manner. The intervention’s development and refinement were informed by community-based research, engaging study participants at each stage to ensure that both study implementation and intervention content adequately address the needs of those requiring assistance.

The multidisciplinary team of nurses, case workers, and social workers in a safety net HIV clinic offers comprehensive services to address adverse SBDOH. In the clinic’s routine operations, patient identification and referral typically occur during scheduled visits or when patients reach out to their primary provider or a member of the multidisciplinary team. Upon activation, the team conducts assessments of SBDOH to address key issues. The clinic’s social workers and case managers are recognized within the community for their ability to address adverse SBDOH, including substance use disorder, housing instability, food insecurity, and other relevant factors. This integrated intervention is highly innovative in that it combines the accuracy from modern IT-based ingestible sensor system for measuring real time ART taking and the driving force from real life SBDOH to timely and effectively address and leverage adherence to medications.

The integration of pre-and post-dose text reminder messaging technology to support adherence is highly acceptable and feasible in the pilot RCT of a prior study conducted by our team and published elsewhere [24, 35]. The implementation of this technology in this study will further enhance the modality of the intervention centered on individual needs and HIV medication patterns.

This study has several potential limitations. First, the decision was made not to implement the ID-Cap technology in the control arm. While using ID-Cap could provide more accurate adherence data, it may alter patients’ behavior and perceptions, thus deviating from the original UC protocol. Therefore, the UC arm will rely on self-reported adherence, which is known to overestimate true adherence and typically provides only an estimate of the percentage of prescribed doses taken. To address this limitation, self-reported adherence data will also be collected in the intervention arm to estimate systematic shifts in adherence compared to ID-Cap adherence. This will help develop calibration models to adjust self-reported adherence using ID-Cap data as the reference. Additionally, supplementary questions on missed doses will be included to better align self-reported adherence with levels observed in the intervention arm. Second, while the domains and content of SBDOH are standardized, the nature and intensity of SBDOH intervention sessions are tailored to each participant. To ensure consistency, standardized curricula and manuals for each social and behavioral determinants of health domain (e.g., risky sexual behavior) at various intensity levels (e.g., low/medium/high) will be developed, facilitating implementation across diverse clinical settings. Third, more stringent inclusion criteria, such as requiring detectable HIV viral loads, were considered but ultimately not adopted due to concerns about feasibility and the relevance of the intervention’s impact on high-risk sexual behavior and SBDOH in individuals with undetectable viral loads. Consequently, all individuals with poor adherence or at high risk for poor adherence, regardless of their viral load status, are included. Lastly, measuring the extent of adverse SBDOH and the frequency and timeliness of intervention sessions may be challenging. To address this challenge, we will employ non-parametric methods (e.g., counting adverse SBDOH events), semi-parametric techniques (e.g., categorizing SBDOH severity as low/medium/high), and fully parametric analyses (e.g., measuring the time until the first SBDOH intervention session) to effectively capture variability in these measures.

In summary, informed by prior work and community collaboration, we developed a multidisciplinary team intervention targeting SBDOH. This study employs a rigorous longitudinal design and will recruit participants from a large safety net HIV clinic in the Los Angeles area, utilizing an advanced ingestible sensor system to trigger real-time intervention sessions upon detecting pre-determined missed doses patterns. The intervention specifically addresses challenges associated with poor adherence and viral suppression. If effective, this enhanced approach could significantly improve adherence to antiretroviral medications in HIV treatment, particularly for individuals struggling with adherence and those at high risk for virologic failure and transmission of HIV.

## Supplementary Information

Supplementary Material 1.

## Data Availability

No datasets were generated or analysed during the current study.

## Abbreviations

ART: Antiretroviral therapy
ARV: Antiretroviral
CART: Classification and regression trees
CDC: Centers for Disease Control and Prevention
CONSORT: Consolidated Standards of Reporting Trials
DOT: Directly observed therapy
GAMM: Generalized additive mixed model
GCP: Good Clinical Practice
GEE: Generalized estimating equation
GLMM: Generalized linear mixed models
IOM: Institute of Medicine
IRB: Institutional review board
ISS: Ingestible sensor system
MEMS: Medication event monitoring system
PLWH: People living with HIV
SBDOH: Social and behavioral determinants of health
SDOH: Social determinants of health
SMS: Short message service
STIs: Sexually transmitted infections
U=U: Undetectable equals Untransmittable
UC: Usual Care
